# In situ IR spectroscopy during oxidation process of cobalt Prussian blue analogues

**DOI:** 10.1038/s41598-021-83699-8

**Published:** 2021-02-18

**Authors:** Hideharu Niwa, Toshiaki Moriya, Takayuki Shibata, Yuya Fukuzumi, Yutaka Moritomo

**Affiliations:** 1 Graduate School of Pure and Applied Sciences, University of Tsukuba, Tsukuba, 305‐8571 Japan; 2 Faculty of Pure and Applied Sciences, University of Tsukuba, Tsukuba, 305‐8571 Japan; 3 Tsukuba Research Center for Energy Materials Science (TREMS), University of Tsukuba, Tsukuba, 305‐8571 Japan; 4 National Institute of Technology, Gunma College, Maebashi, Gunma 371-8530 Japan

**Keywords:** Batteries, Electrochemistry, Thermoelectrics, Coordination polymers

## Abstract

Cobalt Prussian blue analogues (Co-PBA; Na_*x*_Co[Fe(CN)_6_]_*y*_), consisting of cyano-bridged transition metal network, –Fe–CN–Co–NC–Fe–, are promising cathode materials for Na-ion secondary batteries. In the oxidation process, oxidization of Fe and/or Co are compensated by Na^+^ deintercalation. Here, we investigated the oxidization process of three Co-PBAs by means of in situ infrared absorption (IR) spectroscopy. With use of an empirical rule of the frequencies of the CN^−^ stretching mode in ferrocyanide ([Fe^II^(CN)_6_]^4−^) and ferricyanide ([Fe^III^(CN)_6_]^3−^), the oxidation processes of Co-PBAs were determined against the Fe concentration (*y*) and temperature (*T*). We will discuss the interrelation between the oxidation processes and Fe concentration (*y*).

## Introduction

**Figure 1:**
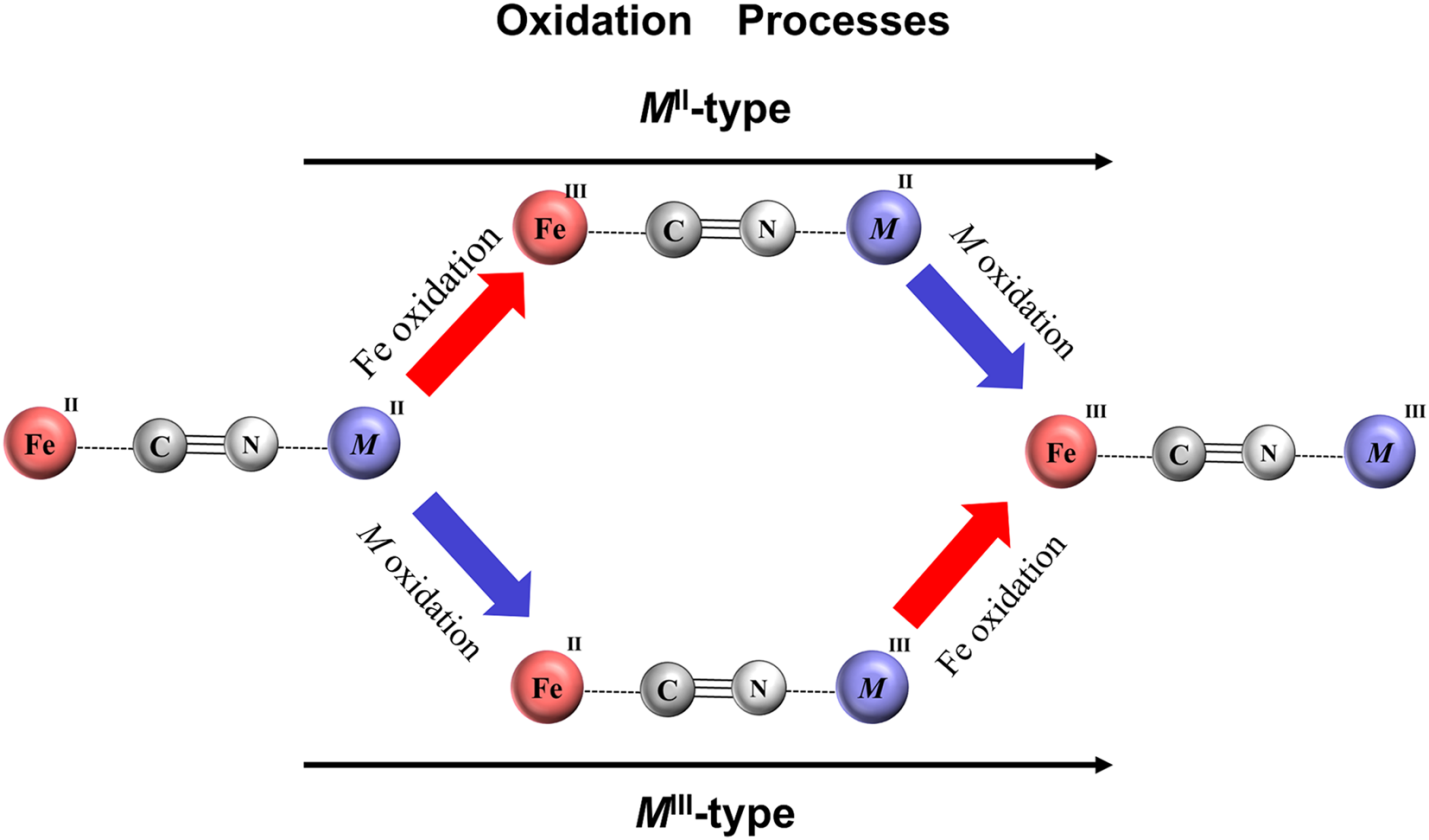
Oxidation processes in *A*_*x*_*M*[Fe(CN)_6_]_*y*_. We call the upper and lower processes as *M*^II^-type and *M*^III^-type, respectively.

Prussian blue analogues (PBA; *A*_*x*_*M*[Fe(CN)_6_]_*y*_) consist of cyano-bridged three-dimensional transition metal network, –*M–*NC–Fe–CN–*M–*NC–Fe–CN–*M–* (*A* and *M* are alkali and transition metals, respectively), and alkali cation located in the nanopores of the network. Among them, cobalt PBA (Co-PBA), represented as Na_*x*_Co[Fe(CN)_6_]_*y*_, have the face-centered cubic (fcc) ($Fm\stackrel{-}{3}m$; *Z* = 4) or trigonal ($R\stackrel{-}{3}m$; *Z* = 3) structures^1–3^. The Co-PBA is one of the promising cathode materials for lithium-ion/sodium-ion secondary batteries^4–14^, because Na^+^ can be electrochemically intercalated into (deintercalated from) the nanopores of the network. The Na^+^ deintercalation accompanies the oxidization of metal ions, *i.e.*, Fe and Co, from the divalent to trivalent states. As schematically shown in Fig. 1, PBA has two kinds of oxidation processes; *M*^II^Fe^II^ → *M*^II^Fe^III^ → *M*^III^Fe^III^ and *M*^II^Fe^II^ → *M*^III^Fe^II^ → *M*^III^Fe^III^, because it has two metal sites. We will call the former (latter) process as *M*^II^-type (*M*^III^-type) because *M* in the intermediate state is divalent (trivalent). The synchrotron-based X-ray absorption near edge structure (XANES) measurements and X-ray photoelectron spectroscopy (XPS) are powerful tool to determine the valence state of transition metals during the oxidization process^8,15–21^. For example, Takachi et al*.* investigated the oxidization process of Na_*x*_Mn[Fe(CN)_6_]_0.83_ (NMF83) and Na_*x*_Co[Fe(CN)_6_]_0.90_ (NCF90), and found that the processes are classified into *M*^II^-type and *M*^III^-type, respectively^8^.

On the other hands, infrared (IR) spectroscopy is a sensitive probe of the CN^-^ stretching mode. Especially, the spectroscopy can clarify the effect of the valence changes of the sandwiching *M* and Fe, i.e., *M–*NC–Fe, on the CN^-^ stretching mode. Several researchers^6,22–31^ have reported the characteristic valence effect on the mode. In Table 1, we summarized the interrelation between the frequency (*ν*_CN_) of the CN^-^ stretching mode and the valence of the neighboring Fe, that is, divalent in ferrocyanide ([Fe^II^(CN)_6_]^4-^) and trivalent ferricyanide ([Fe^III^(CN)_6_]^3-^). Regardless of *A* and *M*, the *ν*_CN_ in ferrocyanide (ferricyanide) is ranged from 2065 to 2135 cm^-1^ (2145 to 2205 cm^-1^). Hereafter, we will call this relation as “empirical rule”. The empirical rule tells us that Fe takes divalent (trivalent) if *ν*_CN_ < 2135 cm^-1^ (*ν*_CN_ > 2145 cm^-1^). On the other hands, Moriya et al. performed in situ IR spectroscopy of Co- and Mn-PBAs^32^. They clarified the persistent and/or amalgamation type behavior of the CN^-^ stretching mode during the oxidization process. The persistent and/or amalgamation type behavior are well classified by magnitude of Δ*/W*, where Δ and *W* are the energy difference between divalent and trivalent states and band width of the CN^-^ stretching mode, respectively. Thus, IR spectroscopy is a sensitive probe for the CN^-^ stretching mode. And, there exists the empirical rule between the Fe valence and *ν*_CN_. Then, we can detect the valence change of Fe and *M* via *ν*_CN_ during the oxidization process_,_ and hence, can determine the type of the oxidization process, i.e. *M*^II^ and *M*^III^-type.

**Table 1:** The frequency (*ν*_CN_) of the CN^-^ stretching mode in [Fe^II^(CN)_6_]^4-^ and [Fe^III^(CN)_6_]^3-^ in Prussian blue analogues (PBA; *A*_*x*_*M*[Fe(CN)_6_]_*y*_).

*M*	*A*	Fe^II^		Fe^III^		References
		Samples	*ν*_CN_ (cm^-1^)	Samples	*ν*_CN_ (cm^-1^)	
Co	–	Co_2_[Fe(CN)_6_]	2085	Co_3_[Fe(CN)_6_]_2_	2160, 2200	^22^
Co	Na	Na_1.4_Co_1.3_[Fe(CN)_6_]	2100	Na_0.4_Co_1.3_[Fe(CN)_6_]	2160	^23^
Co	K	K_0.4_Co_1.3_[Fe(CN)_6_]	2135			^23^
Co	Na	Na_0.84_Co[Fe(CN)_6_]_0.71_	2080	Na_0.23_Co[Fe(CN)_6_]_0.71_	2160	^24^
Co	K	K_0.1_Co_4_[Fe(CN)_6_]_2.7_	2090, 2118	K_0.1_Co_4_[Fe(CN)_6_]_2.7_	2156	^25^
	Rb	Rb_1.8_Co_4_[Fe(CN)_6_]_3.3_	2125			
	Cs	Cs_3.9_Co_4_[Fe(CN)_6_]_3.9_	2120			
Co	–	Co_2_[Fe(CN)_6_]	2080	Co_3_[Fe(CN)_6_]_2_	2156	^26^
	–			Co[Fe(CN)_6_]	2190–2205	
Co	–	Co_3_[Fe(CN)_6_]_2_	2080, 2120	Co_3_[Fe(CN)_6_]_2_	2160	^27^
Co	Rb	Rb_0.4_Co_4_[Fe(CN)_6_]_2.8_	2095	Rb_0.4_Co_4_[Fe(CN)_6_]_2.8_	2159	^28^
Co	–	Co_3_[Fe(CN)_6_]_2_	2091, 2117	Co_3_[Fe(CN)_6_]_2_	2158	^29^
Co	K	K_2_Co[Fe(CN)_6_]	2087	Co_3_[Fe(CN)_6_]_2_	2155	^30^
	K	K_2_Co_3_[Fe(CN)_6_]	2105	Co[Fe(CN)_6_]	2200	
	K	K_0.4_Co_1.3_[Fe(CN)_6_]	2113–2130			
Mn	–	Mn_3_[Fe(CN)_6_]_2_	2066	Mn_3_[Fe(CN)_6_]_2_	2147	^29^
Mn	Na	Na_1.32_Mn[Fe(CN)_6_]_0.83_	2070	Mn[Fe(CN)_6_]_0.83_	2150	^8^
Mn	–	Mn_3_[Fe(CN)_6_]_2_	2070	Mn_3_[Fe(CN)_6_]_2_	2149	^31^

In this paper, we will demonstrate that the in situ IR spectroscopy is a sensitive tool to determine the type of the oxidization process. We investigated the oxidization process of three Co-PBAs with different Fe concentration (*y* = 0.71, 0.81, and 0.90) and temperature (*T* = 293 and 330 K). With use of the empirical rule of the CN^-^ stretching mode, the oxidation processes of Co-PBA were classified into the *M*^II^-type or *M*^III^-type without ambiguity.

## Results

**Figure 2:**
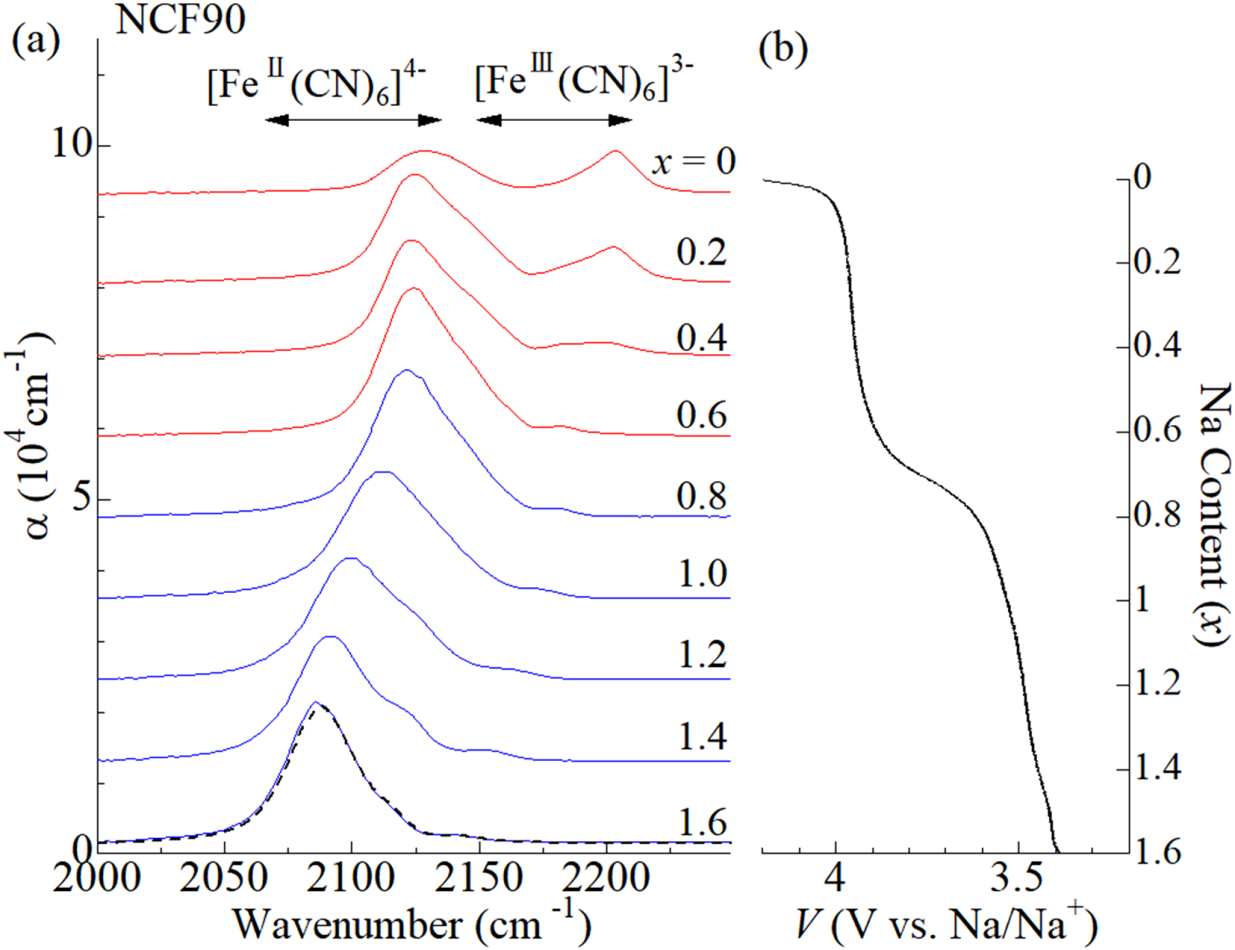
**(a)** In situ IR spectra of the NCF90 film at 293 K against Na concentration (*x*) in the oxidation process from *x* = 1.6 to *x* = 0. Black broken curve at *x* = 1.6 was obtained after the measurement. α is absorption coefficient; $\mathrm{\alpha }=\frac{1}{d}\mathrm{ln}\frac{{I}_{0}}{I}$, where *d* is the film thickness, *I*_0_ is the incident beam intensity, and *I* is the transmitted beam intensity, respectively. Spectra are vertically shifted for clarity. The horizontal arrows indicate the empirical region for the CN^-^ stretching modes in [Fe^II^(CN)_6_]^4-^ and [Fe^III^(CN)_6_]^3-^, respectively. **(b)** Charge curve of NCF90 film at 293 K against *x* at 1.6 C. The voltages of the electrochemical cell were scaled to vs. Na/Na^+^.

Figure 2a shows in situ IR spectra of the NCF90 film measured at 293 K. The horizontal arrows indicate the empirical region for the CN^-^ stretching modes in ferrocyanide ([Fe^II^(CN)_6_]^4-^) and ferricyanide ([Fe^III^(CN)_6_]^3-^), respectively. Figure 2b shows the corresponding charge curve of the NCF90 film against *x* at 1.6 C. The charge curve shows two plateaus at around 3.5 and 4.0 V vs. Na/Na^+^, indicating two step oxidation process. In the lower-lying plateau near 3.5 V, single CN^-^ stretching mode is observed in the range from 2080 cm^-1^ to 2120 cm^-1^. The empirical rule of the CN^-^ stretching mode (horizontal arrows) indicates that the Fe remains divalent in this plateau. Then, the oxidization process in the lower-lying plateau is carried out by the valence change of Co from Co^2+^ to Co^3+^. Reflecting the valence change of Co, the CN^-^ stretching mode shows blue shift with decrease in *x*. In the higher-lying plateau, an additional CN^-^ stretching mode appears at around 2200 cm^-1^. The spectral weight transfers from lower-lying band at 2120 cm^-1^ to the higher-lying band at 2200 cm^-1^ with decrease in *x*. The empirical rule of the CN^-^ stretching mode indicates that the additional mode at 2200 cm^-1^ is due to ferricyanide. In short, the redox site in the higher-lying plateau is Fe, because the divalent and trivalent Fe coexist. Thus, the in situ IR spectroscopy classifies the oxidation process of NCF90 into the *M*^III^-type. The classification is consistent with the literature^8,15^.

**Figure 3:**
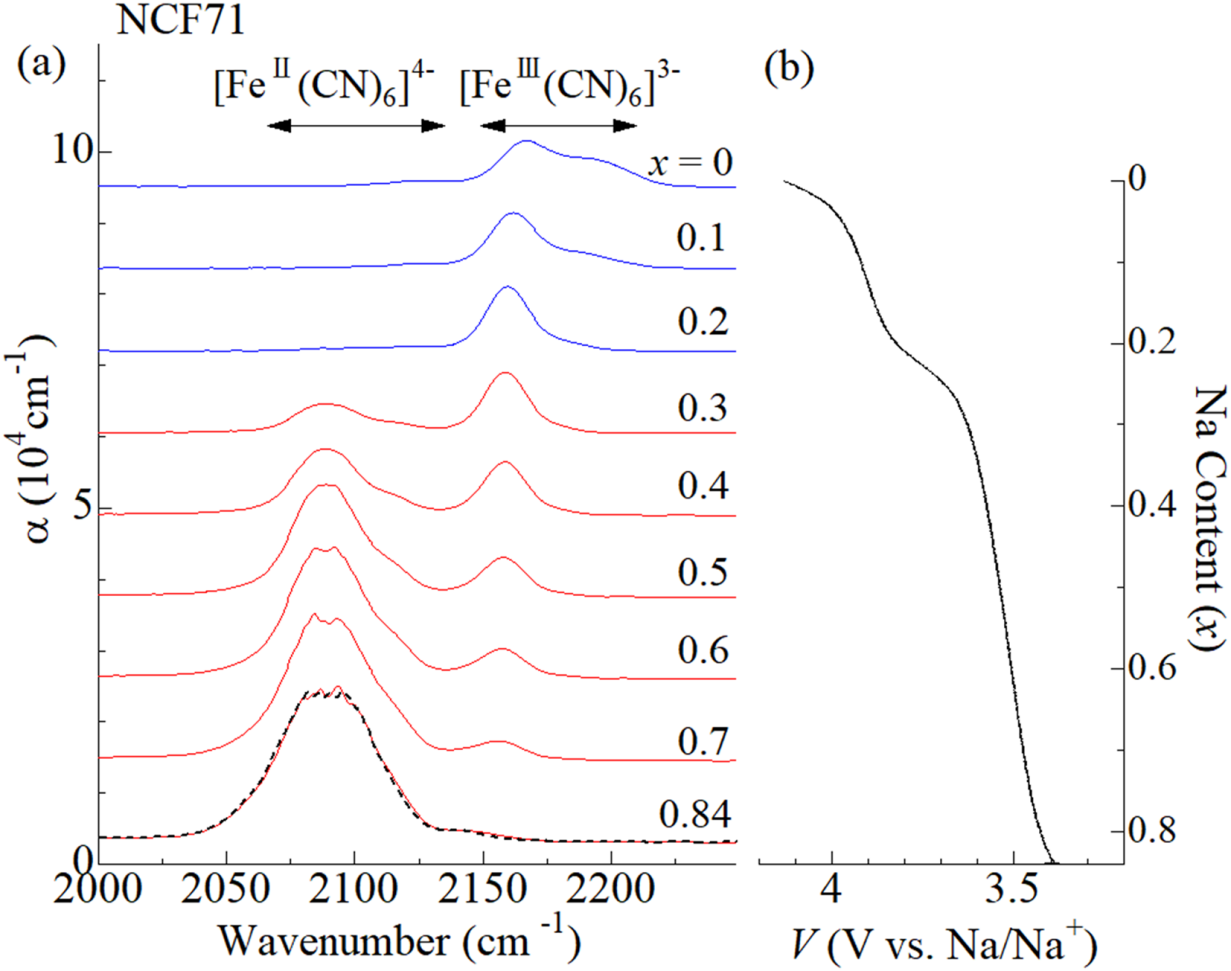
**(a)** In situ IR spectra of the NCF71 film at 293 K against *x* in the oxidation process from *x* = 0.84 to *x* = 0. Black broken curve at *x* = 0.84 was obtained after the measurement. α is absorption coefficient; $\mathrm{\alpha }=\frac{1}{d}\mathrm{ln}\frac{{I}_{0}}{I}$, where *d* is the film thickness, *I*_0_ is the incident beam intensity, and *I* is the transmitted beam intensity, respectively. Spectra are vertically shifted for clarity. The horizontal arrows indicate the empirical region for the CN^-^ stretching modes in [Fe^II^(CN)_6_]^4-^ and [Fe^III^(CN)_6_]^3-^, respectively. **(b)** Charge curve of the NCF71 film at 293 K against *x* at 1.9 C. The voltages of the electrochemical cell were scaled to vs. Na/Na^+^.

Figure 3a shows in situ IR spectra of Na_0.84_Co[Fe(CN)_6_]_0.71_ (NCF71) film measured at 293 K. The horizontal arrows indicate the empirical region for the CN^-^ stretching modes in ferrocyanide ([Fe^II^(CN)_6_]^4-^) and ferricyanide ([Fe^III^(CN)_6_]^3-^), respectively. Figure 3b shows the corresponding charge curve of the NCF71 film against *x* at 1.9 C. The charge curve shows two plateaus at around 3.5 and 4.0 V vs. Na/Na^+^, indicating two step oxidation process. In the lower-lying plateau, two CN^-^ stretching modes are observed in the range at around 2090 cm^-1^ and 2155 cm^-1^. The spectral weight transfers from lower-lying band at 2090 cm^-1^ to the higher-lying band at 2155 cm^-1^ with decrease in *x*. The empirical rule indicates that the former and latter mode are ascribed to the ferrocyanide and ferricyanide, respectively. In short, the redox site in the lower-lying plateau is Fe, because the divalent and trivalent Fe coexist. In the higher-lying plateau, two CN^-^ stretching modes are observed in the range at around 2155 cm^-1^ and 2185 cm^-1^. The empirical rule indicates that the Fe is trivalent in this plateau. Then, the oxidization process in the higher-lying plateau is carried out by the valence change of Co from Co^2+^ to Co^3+^. With decrease in *x*, the spectral weight transfers from the lower-lying to higher-lying bands. Therefore, the lower-lying (higher-lying) band can be ascribed to the Fe^III^-CN-Co^II^ (Fe^III^-CN-Co^III^) mode. Thus, the in situ IR spectroscopy classifies the oxidation process of NCF71 into the *M*^II^-type. The classification is consistent with the literature^23^.

**Figure 4:**
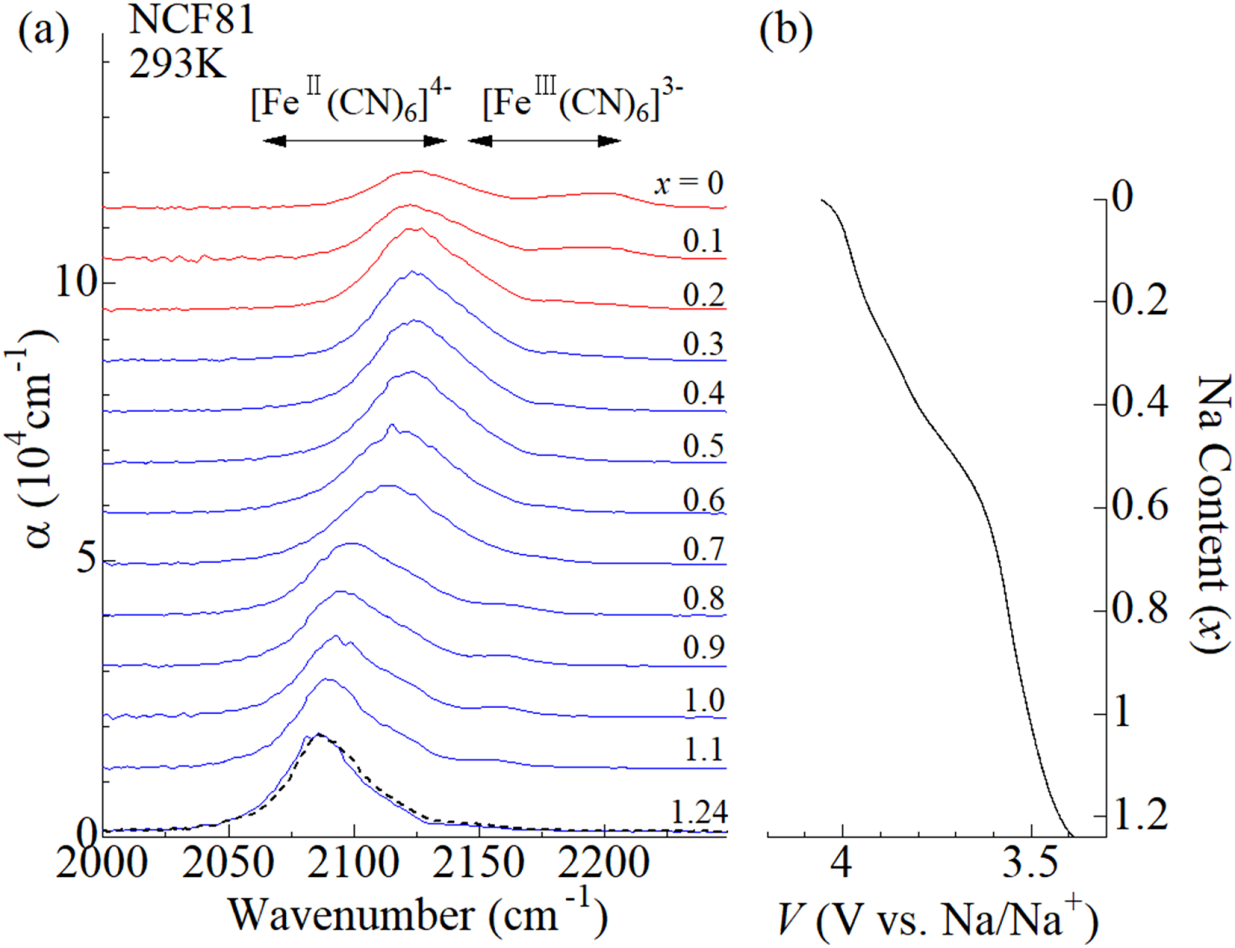
**(a)** In situ IR spectra of the NCF81 film at 293 K against *x* in the oxidation process from *x* = 1.24 to *x* = 0. Black broken curve at *x* = 1.24 was obtained after the measurement. α is absorption coefficient; $\mathrm{\alpha }=\frac{1}{d}\mathrm{ln}\frac{{I}_{0}}{I}$, where *d* is the film thickness, *I*_0_ is the incident beam intensity, and *I* is the transmitted beam intensity, respectively. Spectra are vertically shifted for clarity. The horizontal arrows indicate the empirical region for the CN^-^ stretching modes in [Fe^II^(CN)_6_]^4-^ and [Fe^III^(CN)_6_]^3-^, respectively. **(b)** Charge curve of the NCF81 film measured at 293 K against *x* at 2.0 C. The voltages of the electrochemical cell were scaled to vs. Na/Na^+^.

Figure 4a shows in situ IR spectra of the Na_1.24_Co[Fe(CN)_6_]_0.81_ (NCF81) film measured at 293 K. Figure 4b shows the corresponding charge curve of the NCF81 film against *x* at 2.0 C. The charge curve shows two plateaus at around 3.5 and 3.8 V vs. Na/Na^+^, indicating two step oxidation process. In the lower-lying plateau near 3.5 V, single CN^-^ stretching mode is observed in the range from 2085 cm^-1^ to 2125 cm^-1^. The empirical rule indicates that the Fe is divalent in this plateau. Then, the oxidization process in the lower-lying plateau is carried out by the valence change of Co from Co^2+^ to Co^3+^. Reflecting the valence change of Co, the CN^-^ stretching mode shows blue shift with decrease in *x*. In the higher-lying plateau, an additional CN^-^ stretching mode appears at around 2200 cm^-1^. The spectral weight transfers from lower-lying band at 2125 cm^-1^ to the higher-lying band at 2200 cm^-1^ with decrease in *x*. The empirical rule indicates that the additional mode at 2200 cm^-1^ is due to ferricyanide. In short, the redox site in the higher-lying plateau is Fe, because the divalent and trivalent Fe coexists. Thus, the in situ IR spectroscopy classifies the oxidation process of NCF81 at 293 K into the *M*^III^-type.

**Figure 5:**
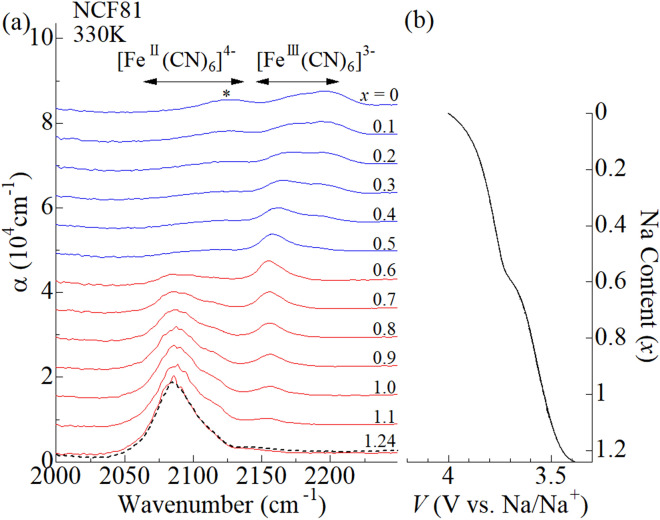
**(a)** In situ IR spectra of the NCF81 film at 330 K against *x* in the oxidation process from *x* = 1.24 to *x* = 0. Black broken curve at *x* = 1.24 was obtained after the measurement. α is absorption coefficient; $\mathrm{\alpha }=\frac{1}{d}\mathrm{ln}\frac{{I}_{0}}{I}$, where *d* is the film thickness, *I*_0_ is the incident beam intensity, and *I* is the transmitted beam intensity, respectively. Spectra are vertically shifted for clarity. The horizontal arrows indicate the empirical region for the CN^-^ stretching modes in [Fe^II^(CN)_6_]^4-^ and [Fe^III^(CN)_6_]^3-^, respectively. Origin of the band marked by asterisk is unknown. **(b)** Charge curve of the NCF81 film at 330 K against *x* at 1.4 C. The voltages of the electrochemical cell were scaled to vs. Na/Na^+^.

Figure 5a shows in situ IR spectra of the NCF81 film at 330 K. We note that the *x*-dependent spectral changes of the NCF81 film are qualitatively different between at 293 K (Fig. 4a) and at 330 K (Fig. 5a). Figure 5b shows the corresponding charge curve of the NCF81 film at 1.4 C. The charge curve shows two plateaus at around 3.6 and 3.8 V vs. Na/Na^+^, indicating two step oxidation process. In the lower-lying plateau near 3.6 V, two CN^-^ stretching modes are observed in the range at around 2090 cm^-1^ and 2155 cm^-1^. The spectral weight transfers from lower-lying band at 2090 cm^-1^ to the higher-lying band at 2155 cm^-1^ with decrease in *x*. The empirical rule indicates that the former and latter mode are ascribed to the ferrocyanide and ferricyanide, respectively. In short, the redox site in the lower-lying plateau is Fe, because the divalent and trivalent Fe coexists. In the higher-lying plateau near 3.8 V, two CN^-^ stretching modes are observed in the range at around 2155 cm^-1^ and 2195 cm^-1^. The empirical rule indicates that the Fe is trivalent in this plateau. Then, the oxidization process in the higher-lying plateau is carried out by the valence change of Co from Co^2+^ to Co^3+^. With decrease in *x*, the spectral weight transfers from the lower-lying to higher-lying band. Therefore, the lower-lying (higher-lying) band can be ascribed to the Fe^III^–CN–Co^II^ (Fe^III^–CN–Co^III^) mode. Thus, the in situ IR spectroscopy classifies the oxidation process of NCF81 at 330 K into the *M*^II^-type.

## Discussion

We investigated the redox process of four (*y*, *T*) points, i.e., (0.71, 293 K), (0.81, 293 K), (0.90, 293 K), and (0.81, 330 K), where *y* is the Fe concentration. The IR spectroscopy revealed that (*y*, *T*) = (0.71, 293 K) and (0.81, 330 K) points are *M*^II^-type, and (*y*, *T*) = (0.81, 293 K) and (0.90, 293 K) points are *M*^III^-type. In the *y*–*T* plane, the *M*^II^-type reaction appears when the *T* is raised or *y* is lowered from the (0.81, 293 K) point.

The Co-PBA in the intermediate state shows a phase transition from low-spin (LS) to high-spin (HS) phases with increase in temperature^33,34^. The electronic configuration is LS Co^3+^ and LS Fe^2+^ in the LS phase, while it is HS Co^2+^ and LS Fe^3+^ in the HS phase. The phase transition accompanies cooperative charge transfer from Fe^2+^ to Co^3+^ as well as the expansion of the unit cell volume. The volume expansion is ascribed to the larger ionic radius of HS Co^2+^. We consider that the relative stability of the two phases in the intermediate state determines the type of the oxidization process; the oxidization process is *M*^III^-type if the LS phase is stable, while the oxidization process is *M*^II^-type if the HS phase is stable. The HS state, and hence, the *M*^II^-type process, is stabilized with decrease in *y*, because the weaker ligand field from the octahedrally coordinated [Fe(CN)_6_] stabilizes the HS Co^2+^ configuration. This argument well explains why the *M*^II^-type reaction appears when the *T* is raised, or *y* is lowered.

## Summary

We demonstrated that the in situ IR spectroscopy is a sensitive tool to determine the type of the oxidization process. The oxidation processes were classified into the *M*^II^-type or *M*^III^-type against Fe concentration (*y*) and temperature; The *y* = 0.71 (at 293 K) and 0.81 (at 330 K) compounds are classified into *M*^II^-type while the *y* = 0.81 (at 293 K) and 0.90 (at 293 K) compounds are classified into *M*^III^-type. Especially, the *y* = 0.81 shows thermal switching of the oxidation process from the *M*^III^-type (293 K) to *M*^II^-type (at 330 K). Such a thermal switching is advantageous for thermal energy harvesting, as demonstrated by Shibata et al.^35^ in the tertiary battery with use of phase transition.

## Methods

### Sample preparation

Thin films of Na_1.60_Co[Fe(CN)_6_]_0.9_ (NCF90), Na_1.24_Co[Fe(CN)_6_]_0.81_ (NCF81), Na_0.84_Co[Fe(CN)_6_]_0.71_ (NCF71), and Na_0.72_Ni[Fe(CN)_6_]_0.68_ (NNF68) were synthesized by electrochemical decomposition method. The films were deposited on the indium tin oxide (ITO) transparent electrode coated on cover glass substrate under potentiostatic conditions at − 0.45 V vs. the Ag/AgCl electrode. The electrolytes for the NCF90 film^36^ were an aqueous solution containing 0.8 mmol/L K_3_[Fe(CN)_6_], 0.5 mmol/L Co(NO_3_)_2_, and 5.0 mol/L NaNO_3_. The electrolytes for the NCF81 film^35^ were an aqueous solution containing 0.8 mmol/L K_3_[Fe(CN)_6_], 0.5 mmol/L Co(NO_3_)_2_, and 0.5 mol/L NaNO_3_. The electrolytes for the NCF71 film^24^ were an aqueous solution containing 0.5 mmol/L K_3_[Fe(CN)_6_], 1.25 mmol/L Co(NO_3_)_2_, and 1.0 mol/L NaNO_3_. The electrolytes for the NNF68 film were an aqueous solution containing 0.5 mmol/L K_3_[Fe(CN)_6_], 0.5 mmol/L Ni(NO_3_)_2_, and 1.0 mol/L NaNO_3_. Synthesis condition of the PBA films are summarized in Table 2. In this process, the reduction reaction ([Fe(CN)_6_]^3-^ + e^-^ → [Fe(CN)_6_]^4-^) triggers the deposition of PBAs. Therefore, Fe, Co and Ni in the as-grown films are divalent. Among the three Co-PBA film, NCF90 film shows the highest discharge capacity (135 mAh/g)^8^. In addition, the NCF90 film exhibits excellent rate properties (discharge capacity at 60C is 90% of the OCV value)^8^.

**Table 2:** Synthesis condition of PBA films by electrochemical decomposition method.

Samples	Electrolytes	Concentrations	Deposition time
NCF90	K_3_[Fe(CN)_6_]	0.8 mmol/L	30 min
	Co(NO_3_)_2_	0.5 mmol/L	
	NaNO_3_	5.0 mol/L	
NCF81	K_3_[Fe(CN)_6_]	0.8 mmol/L	15 min
	Co(NO_3_)_2_	0.5 mmol/L	
	NaNO_3_	0.5 mol/L	
NCF71	K_3_[Fe(CN)_6_]	0.5 mmol/L	15 min
	Co(NO_3_)_2_	1.25 mmol/L	
	NaNO_3_	1.0 mol/L	
NNF68	K_3_[Fe(CN)_6_]	0.5 mmol/L	45 min
	Ni(NO_3_)_2_	0.5 mmol/L	
	NaNO_3_	1.0 mol/L	

The X-ray diffraction patterns of the NNF68, NCF71, NCF81, and NCF90 films at 293 K were shown in Figure S1. The X-ray source was the Cu Kα line. NNF68, NCF71, and NCF81 show face-centered cubic (fcc) ($Fm\stackrel{-}{3}m$; *Z* = 4) structure. The lattice constants (*a*) of NNF68, NCF71, and NCF81 are 10.15 Å, 10.26 Å, and 10.26 Å, respectively. NCF90 shows trigonal ($R\stackrel{-}{3}m$; *Z* = 3) structure. The lattice constants *a* and *c* of NCF90 are 7.40 Å and 17.49 Å. The chemical compositions of the films were determined by the inductively coupled plasma (ICP) method and CHN organic elementary analysis (PerkinElmer 2400 CHN Elemental Analyzer). Thickness of the NCF90, NCF81, and NCF71 films were 1.36 μm, 1.39 μm, and 1.93 μm, respectively, which were evaluated by a profilometer (aep Technology NanoMap-LS).

### In situ infrared spectroscopy

The in situ IR spectra were measured with the use of an infrared microscopy system (JASCO IRT-3000) equipped with a Fourier transform infrared spectrometer (JASCO FT/IR-660 Plus) in the 400–7000 cm^-1^ region with 2 cm^-1^ resolution. The transmitted light was focused on a cooled HgCdTe detector. The Na concentrations (*x*) were electrochemically controlled in the oxidation (charging) process with a potentiostat (Bio-logic VSP multichannel potentiostat). *x* in NCF90, NCF81, and NCF71 films were evaluated from the extracted charge under the assumption that *x* = 0.0 in the fully oxidized state. In situ IR spectra of NCF71, NCF81, and NCF90 at 293 K were measured in the first oxidation process films. The spectra of NCF81 at 330 K were measured in the second oxidation process films after the measurement at 293 K. We confirmed that the spectral profiles returned to the initial ones after the reduction process. Each spectrum was recorded in every 90 s during the oxidization process.

### Optical electrochemical cell

**Figure 6:**
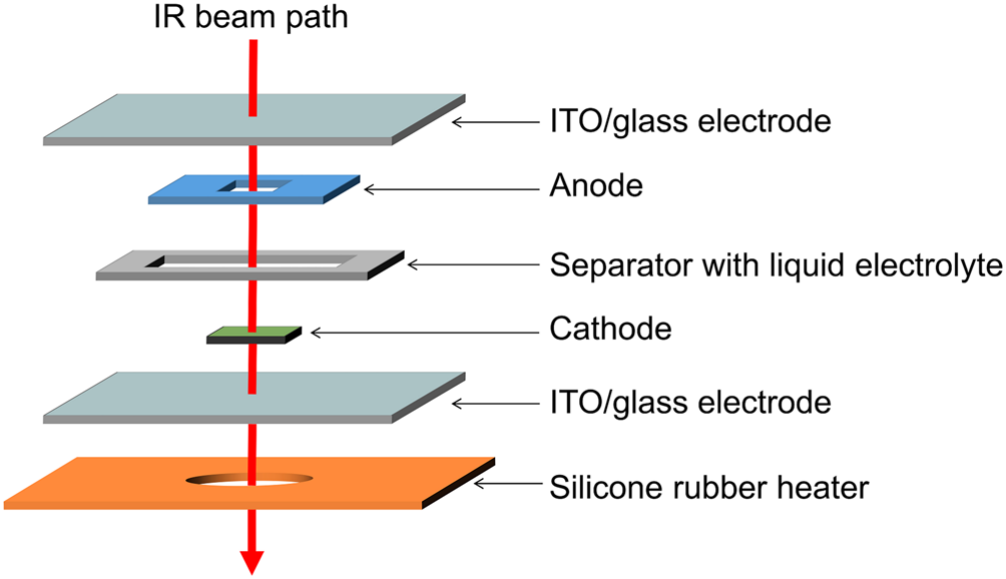
Schematic representation of the optical electrochemical cell for in situ IR measurements.

Figure 6 schematically shows the optical electrochemical cell used for the in situ IR measurements. The cathode, anode and electrolyte were the Co-PBA and NNF68 film grown on the ITO glass substrates, and 17 mol/kg NaClO_4_ aqueous solution. To measure the IR spectra of the Co-PBA film, the center of NNF68 film has hollow. The anode and cathode sandwich a separator whose thickness was 25 μm. The voltage between the anode and cathode was controlled so that a constant current (75 μA/cm^2^) would flow between the electrodes. The voltage control range was from − 0.05 V to 1.0 V vs. NNF68. The cell temperature was controlled by a silicone rubber heater. IR absorptions due to the ITO glass substrates and the electrolyte were subtracted as backgrounds.

We chose the NNF68 film as anode, because the film is stable in aqueous electrolyte^37^. The anode NNF68 were pre-oxidized to 3.45 V vs. Na/Na^+^. The active area of anode (213 mm^2^) was set to be much larger than that of cathode (4 mm^2^) so that *x*, and hence, the potential of anode can be regarded as constant, as schematically shown in Fig. S2. Therefore, the voltage vs. Na/Na^+^. can be obtained by adding 3.45 V to the voltage vs. NNF68 in the optical cell. In the in situ IR experiment, the current density was determined so that the charge rate was 1 C (= 75 μA/cm^2^), assuming that the film thickness was 1 μm. After the measurement, the film thickness was determined with use of the profilometer. Mass was evacuated from the thickness and area (= 4 mm^2^). Mass of the NCF90, NCF81, and NCF71 films were 5.8 μg, 6.6 μg, and 6.7 μg, respectively. The actual charge rate of NCF90 at 293 K, NCF81 at 293 K, NCF81 at 330 K, and NCF71 at 293 K were 1.6 C, 2.0 C, 1.4 C, and 1.9 C, respectively.

## Supplementary Information

Supplementary Figures.
