# Supplementary material for: In situ IR spectroscopy during oxidation process of cobalt Prussian blue analogues
Source: Sci Rep. 2021 Feb 18;11:4119. doi: 10.1038/s41598-021-83699-8 (PMC7892820; doi:10.1038/s41598-021-83699-8)
Supplement: Supplementary file 1 — Supplementary Figures. [file 41598_2021_83699_MOESM1_ESM.docx]

Supplementary information

*In situ* IR spectroscopy during oxidation process of cobalt Prussian Blue analogues

Hideharu Niwa^1‐3^*, Toshiaki Moriya^1^, Takayuki Shibata^4^, Yuya Fukuzumi^1^, Yutaka Moritomo^1‐3^*

^1^Graduate School of Pure and Applied Sciences, University of Tsukuba, Tsukuba 305‐8571, Japan

^2^Faculty of Pure and Applied Sciences, University of Tsukuba, Tsukuba 305‐8571, Japan

^3^Tsukuba Research Center for Energy Materials Science (TREMS), University of Tsukuba, Tsukuba 305‐8571, Japan

^4^National Institute of Technology, Gunma College, Maebashi, Gunma, 371-8530, Japan

E-mail: niwa.hideharu.ga@u.tsukuba.ac.jp, moritomo.yutaka.gf@u.tsukuba.ac.jp


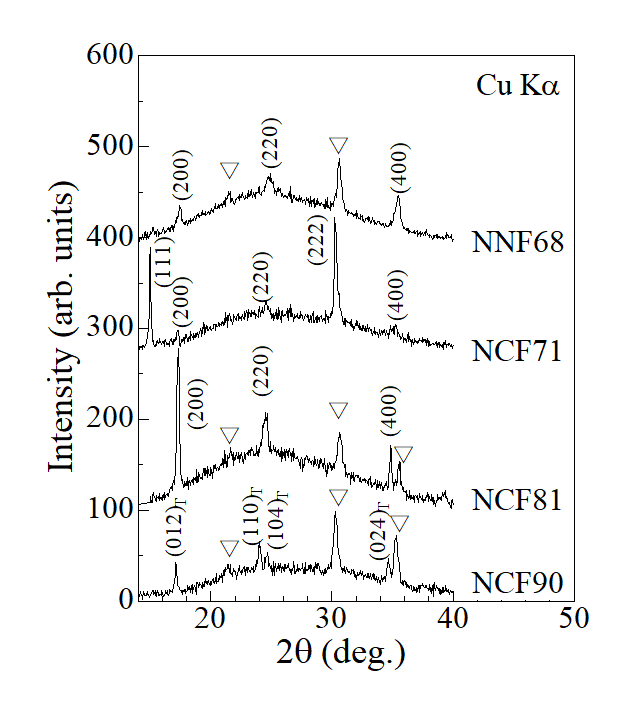


**Figure S1**. X-ray diffraction patterns of the NNF68, NCF71, NCF81, and NCF90 films at 293K. The X-ray source was the Cu Kα line. NNF68, NCF71, and NCF81 show face-centered cubic (fcc) ($Fm\bar{3}m$; *Z* = 4) structure. Numbers in parentheses represent the index in the cubic cell. NCF71 film showed (111) orientation. NCF90 shows trigonal ($R\bar{3}m$; *Z* = 3) structure. Numbers in parentheses represent the index in the trigonal cell (hexagonal setting). Triangles represent diffraction peaks due to ITO.

**Figure S2**. (a) Charge curve of NNF68 film (213 mm^2^) at 293K. The charge rate was 1.3 C. (b) Charge curve of NCF90 (4 mm^2^) film at 293K. The charge rate was 1.6 C. The NCF90 and NNF68 films were used as the cathode and anode in the electrochemical cell for *in situ* IR measurements. Horizontal arrows represent the voltage change in the oxidization process of NCF90. The voltage change in the NNF68 film is less than 17 mV.
